# Challenges and next steps in the advancement of immunotherapy: summary of the 2018 and 2020 National Cancer Institute workshops on cell-based immunotherapy for solid tumors

**DOI:** 10.1136/jitc-2021-003048

**Published:** 2021-07-15

**Authors:** Laura K Fogli, Rosemarie Aurigemma, Connie L Sommers, Anju Singh, Kasia Bourcier, Marc S Ernstoff, NCI Cell Therapy Workshop Committee, Steven Albelda

**Affiliations:** 1 ImmunoOncology Branch, Developmental Therapeutics Program, Division of Cancer Treatment and Diagnosis, National Cancer Institute, Rockville, Maryland, USA; 2 Office of the Associate Director, Developmental Therapeutics Program, Division of Cancer Treatment and Diagnosis, National Cancer Institute, Rockville, Maryland, USA

**Keywords:** immunotherapy, adoptive, T-lymphocytes, receptors, chimeric antigen, lymphocytes, tumor-infiltrating, cell therapy

## Abstract

Cell-based immunotherapies have had remarkable success in the clinic, specifically in the treatment of hematologic malignancies. However, these strategies have had limited efficacy in patients with solid tumors. To better understand the challenges involved, the National Cancer Institute (NCI) convened an initial workshop with immuno-oncology thought leaders in December 2018 and a follow-up workshop in December 2020. The goals of the NCI workshops on cell-based immunotherapy for solid tumors were to discuss the current state of the field of cell-based immunotherapy, obtain insights into critical knowledge gaps, and identify ways in which NCI could facilitate progress. At both meetings, subjects emphasized four main types of challenges in further developing cell-based immunotherapy for patients with solid tumors: scientific, technical, clinical, and regulatory. The scientific barriers include selecting appropriate targets, ensuring adequate trafficking of cell therapy products to tumor sites, overcoming the immunosuppressive tumor microenvironment, and identifying appropriate models for these investigations. While mouse models may provide some useful data, the majority of those that are commonly used are immunodeficient and unable to fully recapitulate the immune response in patients. There is therefore a need for enhanced support of small early-phase human clinical studies, preferably with adaptive trial designs, to provide proof of concept for novel cell therapy approaches. Furthermore, the requirements for manufacturing, shipping, and distributing cell-based therapies present technical challenges and regulatory questions, which many research institutions are not equipped to address. Overall, workshop subjects identified key areas where NCI support might help the research community in driving forward innovation and clinical utility: 1) provide focused research support on topics such as tumor target selection, immune cell fitness and persistence, cell trafficking, and the immunosuppressive tumor microenvironment; 2) support the rapid translation of preclinical findings into proof of concept clinical testing, harmonize clinical trial regimens, and facilitate early trial data sharing (including negative results); 3) expand manufacturing support for cell therapies, including vectors and reagents, and provide training programs for technical staff; and 4) develop and share standard operating procedures for cell handling and analytical assays, and work with the Food and Drug Administration to harmonize product characterization specifications.

## Introduction

The expanded knowledge of the immune system over the past half century, coupled with advances in cellular and molecular technologies, has led to a revolution in immunotherapy for cancer. Immunotherapy is now an important and legitimate component of the cancer therapeutic armamentarium. The field overall has seen a rapid surge in research and development across government, industry, and academic institutions, and the remarkable therapeutic potential calls for additional investment to accelerate progress and expand treatment options for patients with cancer.

Cell-based immunotherapies are one approach that has had tremendous success in the clinic, specifically in the treatment of patients with hematologic malignancies. This class of therapy, known as adoptive cellular therapy (ACT), can be further divided into several subtypes, among them: (1) chimeric antigen receptor (CAR) T-cell therapy; (2) tumor-infiltrating lymphocyte (TIL) therapy; (3) engineered T-cell receptor (TCR)-T-cell therapy; and (4) natural killer (NK) cell and dendritic cell therapies. CAR T-cell therapies are the best studied and most widely known, as several have been approved by the Food and Drug Administration (FDA) for the treatment of patients with non-Hodgkin’s lymphomas and leukemias.^1^ However, other strategies may be required to effectively treat solid tumors. While there is excitement among the cancer research community regarding cell-based therapies, progress in the field faces scientific and technical challenges, including the highly complex regulatory process involved in the development and commercialization of cellular products.

The National Cancer Institute (NCI) has a long history of supporting the cancer immunology research community in basic, translational, and clinical studies through grants and clinical trial networks, and while a number of ACT-related projects are already being funded, NCI aims to identify specific areas in which the research community may require additional support or resources. To identify areas of need in cell-based immunotherapy for solid tumors and further drive developments, the NCI at National Institutes of Health (NIH) convened an initial workshop on 10 December 2018 and 11 December 2018, and then a follow-up workshop on the 2-year anniversary, to engage thought leaders in the immuno-oncology field in in-depth scientific discussion. These 2-day meetings brought together extramural academic researchers, industry scientists, FDA representatives, and NCI staff, allowing NCI to gain insight on major challenges and future directions in the field.

At both workshops, the agenda consisted of several sessions featuring talks by leading experts in the cellular therapy field, followed by extensive panel discussions allowing for interactive dialog between workshop subjects and NCI staff (see online supplemental tables 1 and 2). This report summarizes the critical barriers facing cell therapy researchers, as well as potential ways in which NCI can better support the extramural community in addressing these challenges.

10.1136/jitc-2021-003048.supp1 Supplementary data

## Barriers to progress

### Scientific challenges

Based on discussion at both the 2018 and 2020 workshops, one of the major scientific challenges in developing cell-based immunotherapy approaches for solid tumors is the identification of appropriate targets for therapy. Unlike lineage markers expressed by hematologic malignancies, markers expressed on solid tumors are less tumor-specific and cannot be targeted without possible damage to essential tissues, increasing the risk of toxicity.^2^ Further studies are needed to better understand what level of expression is acceptable in normal tissue, and what level of expression is required by the tumor, for a target to be suitable.

Potential targets fall into four broad categories: overexpressed molecules, cancer testis antigens, neoantigens, and viral antigens.^3^ Continued development of screening approaches as well as approaches to modify in vivo expression will enhance the identification of targets for solid tumors. The development of each series of targets and their associated CAR/TCR constructs entails validation, testing, and selection, which requires significant infrastructure and cost, both of which continue to be barriers for development.

Target selection and the likelihood of toxicity are also dependent on the cell-based strategy being used, with each strategy having advantages and posing challenges.^4^ For example, TILs isolated from a patient’s tumor can be cultured and expanded based on their ability to recognize tumor neoantigens or cancer testis antigens; while selected TILs have the benefit of patient-specific and tumor-specific targeting, the approach is technically challenging because it requires a fresh tumor sample that cannot always be obtained and which does not always yield TILs for expansion.^5 6^ The generation of autologous CAR-T cells, on the other hand, does not rely on the patient’s tumor tissue, but does require the identification of a cancer-associated surface antigen (CAR-T cells do not use human leukocyte antigen presentation and therefore cannot target intracellular antigens). TCR-T cells are engineered to be highly reactive against extracellular *or* intracellular tumor antigens—but engineered TCR-T cells can recognize only proteins, not non-protein antigens such as lipids that rely on different antigen-presenting molecules.^7^ Non-T cell strategies using allogeneic donor cells, such as NK cell therapies, are in development but come with their own challenges related to cell expansion and persistence.^8^

For any cell-based therapy, once a suitable target is characterized and selected, success depends on cells trafficking to the area of the tumor, having sufficient access to tumor cells, and remaining functional. This has presented obstacles as well, as tumor penetration may be hindered by tumor architecture, and T-cell persistence and function may be inhibited in an immunosuppressive tumor microenvironment. Workshop subjects agreed that efforts to improve tumor trafficking and penetration, and non-invasive methods to measure these characteristics, should be prioritized. For example, novel approaches such as in vivo **positron emission tomography** (**PET**) and single-photon emission computerized tomography (SPECT)/CT probes, MRI tracer agents, metabolic profiling, and reporter gene integration may allow for enhanced understanding of trafficking and persistence of cells and their functional state while in the body.

Another priority in cell-based immunotherapy research is finding ways to improve antigen spreading, also known as epitope spreading. Transferred T cells promote an immune response by recognizing their programmed antigen, creating an inflammatory microenvironment, and driving immune cell recruitment. The release of new antigens, which occurs as a result of inflammation and tissue damage, and presence of new immune cell populations allow for responses to be mounted against additional antigens, broadening the immune response against the tumor.^9^ Further studies are needed to determine how the type of cell therapy used may affect the extent of antigen spreading, and how treatment strategies can potentially be combined to optimize antitumor immunity.^10–13^

### Technical challenges

Autologous cell-based therapies are not ‘off-the-shelf’ products like conventional drug-based treatments—manufacturing genetically engineered T-cell products is a complex process with multiple steps. Generally, the procedure includes the following stages, each of which poses its own difficulties and has possibilities for failure: collection of blood from the patient or donor, T-cell isolation, gene modification, T-cell expansion, harvesting, and final product testing. After the initial blood collection, manipulation of the patient material is necessary to separate T cells from other immune cells and obtain a pure T-cell culture, a process that can influence the functionality of the end product. The cell culture media used, metabolic requirements, and spatial architecture of the ex vivo conditions are all critical factors that can alter T-cell phenotype. At the end of the genetic modification and cell expansion process, harvesting of the genetically engineered cells is often a rate-limiting step, as it may be labor-intensive or use automated instrumentation with limited capacity. Furthermore, the time taken to process large cell volumes may be damaging to the cells. Harvested cells must undergo final product release testing, including testing for sterility, efficiency of transduction, and independent growth characteristics, before they can be transferred into patients. The release testing processes require 7–14 days to complete, during which time the cells are typically cryopreserved, introducing additional quality control (QC) issues.^14 15^

There remains great variability in manufacturing processes and platforms among different research institutions, as well as in the starting material collected from patients, highlighting the need for standard characterization of final cell products. Further research is needed to better define optimal quality attributes for final products and to better understand how ex vivo culture conditions affect cell fitness. Methods to maintain the appropriate state of activation for the cell product, reduce apoptosis of the product, and prevent T-cell exhaustion are critical. Predictive markers or correlates of ex vivo cell expansion and in vivo fitness and persistence are also needed.

In addition to these standardization challenges, cell therapy manufacturing presents logistical problems for many institutions. The process requires sufficient space compliant with good manufacturing practice (GMP) regulations, which can be a major limitation. Limited supply of GMP-grade transduction vectors and other critical reagents, as well as the specialized assays needed for product testing, are barriers to accelerating research efforts in ACT. Furthermore, to be performed successfully, the cell therapy manufacturing process requires a highly skilled, specially trained technical workforce. Finding, training, and retaining the necessary staff has been a significant challenge in the cell-based therapy field.

At the 2020 workshop, subjects also emphasized that the development of novel platforms and manufacturing systems for ACT production would significantly enhance the field. There is tremendous opportunity for use of the newest gene-editing technologies that can add to or replace viral-based gene delivery, such as CRISPR/dsDNA, CRISPR/adeno-associated viral (AAV) vectors, transposon-based, and **transcription activator**-**like effector nucleases** (**TALEN**)-based platforms.^16–18^ With these new technologies, there is a gap in harmonizing the studies needed for Investigational New Drug (IND) application submission and a need for streamlining the regulatory process. As multigene editing has already begun and will grow, new technologies will be needed to enhance cell selection for targeted high-efficiency gene manipulation compatible with GMP regulations.

### Clinical and regulatory challenges

Beyond the scientific and technical obstacles involved in designing and manufacturing T-cell based immunotherapies, researchers also face clinical challenges in testing these treatments. Meeting attendees agreed that while animal models are useful for preliminary testing of ACT, the ability of preclinical models to predict how ACT products will behave in human patients is limited. There is therefore a need for enhanced support of early-phase clinical studies in small patient cohorts (as low as 10 patients) to provide proof of principle for new cell therapy approaches. While serious toxicities associated with a CAR-T cell product will often become apparent even within the first cohort of two or three patients, evidence of efficacy may require higher enrollment. More flexible trial designs that allow for adaptive testing of various treatment regimens and combinations more efficiently would help to move the field forward. For multicenter studies, it is critical that there is harmonization in the establishment of starting doses, rules for dose escalation, and grading of adverse events, as well as the ability to accurately capture and share patient data.

Because of the complex multistep manufacturing process required for cell-based therapies, regulatory oversight of these agents is more complex than that for off-the-shelf drugs. The FDA must evaluate all stages of the supply chain, ranging from acquisition of the source material to final product testing, storage, and dosing. Source material is limited, and when using autologous cells, the patient’s health status may allow for only limited time between collection and delivery of the final product. Assessing the potency of the final product requires defining a clear metric, which is difficult with a cell-based therapy; evaluating multiple parameters, such as cell killing and cytokine production, may be most appropriate. The high variability of cell-based treatments further complicates the FDA’s assessment.

With the rise of new gene modification technologies for ACT products, researchers face new challenges regarding required safety testing. While current evidence suggests that the likelihood of malignant transformation of genetically modified cells is extremely low, the full risk is not yet known. The FDA therefore requires use of specific growth assays, which can lead to costly and time-consuming delays in bringing products to clinical testing. As manufacturing methods for cell therapy products evolve, it will be necessary for the research community to review these assays with the FDA to determine their predictive value and develop appropriate testing strategies.

## Recommendations for NCI

The preliminary successes of cell-based therapy have established this approach as one of the more promising strategies to reduce the burden of cancer, but there are significant impediments to overall success in cell therapy development and commercialization. NCI is in a position to address many of these challenges and has indeed made significant progress since the 2018 workshop in providing key resources to the scientific community (table 1). Additional ways in which NCI could contribute to advancement in the field are summarized in the discussion below and in box 1.

**Table 1:** Current NCI resources for cell therapy research

Resource for extramural investigators	How to access
Manufacturing of cell therapy products in a centralized GMP facility	Through the NCI Experimental Therapeutic (NExT) Program or the NCI Cancer Therapy Evaluation Program (CTEP)
Manufacturing of GMP lentiviral and retroviral vectors for cell therapy production	
Standard operating procedures (SOPs) for cell handling, shipment and storage, and analytical assays	On the FNLCR website at: https://frederick.cancer.gov/Science/Bdp/documents/Request.aspx

FNLCR, Frederick National Laboratory for Cancer Research; GMP, good manufacturing practice; NCI, National Cancer Institute.

Box 1 **Recommendations for NCI efforts**

- Provide funding support for:
  - Advancing novel approaches for cell manufacturing (new cell expansion methods, genetic engineering including multigene engineering, alternatives to retroviral-based gene delivery, optimization of closed system manufacturing, new strategies for cell product screening, and so on).
  - Preclinical and translational research to advance cell therapy for solid tumors (tumor targets, immune cell fitness and persistence, cell trafficking, the immunosuppressive tumor microenvironment, development of animal models, and so on).
  - Clinical trials, specifically small proof of concept studies to rapidly gain knowledge of promising new treatment approaches.
  - Development of biomarkers and imaging-based detection of response to therapy.
- Establish core laboratory for characterization of manufactured cell products from extramural investigators.
- Provide QC testing for cell therapy-related reagents (eg, GMP vectors) needed for manufacturing.
- Develop and distribute clinical trial protocol templates.
- Provide investigators with guidance on preparing IND submissions.
- Facilitate communication and knowledge sharing between extramural investigators and the FDA, and establish a dialog to address regulatory issues (ie, harmonization of product characteristic specifications, reevaluation of required testing, and streamlining of IND-enabling activities and clinical trials).
- Provide specialized training for laboratory personnel.

### Recommendations for basic science and technical development

The consensus at both workshops was that NCI should strengthen basic science research and technological advancements for cell therapies through targeted support, such as through issuance of grant Requests for Applications (RFAs). Since the initial workshop, NCI has started to provide targeted support in this area, with supplemental funding awarded to NCI-designated cancer center grants and Specialized Programs of Research Excellence (SPORE) grants in 2019 for research projects promoting greater efficacy and broad-based adoption of cell-based therapies (https://dctd.cancer.gov/NewsEvents/20190923_nci_announces_support.htm). Areas of basic science research that continue to be high-priority in the field include the identification of ideal tumor target characteristics, the study of immune cell fitness and persistence, the reversal of immunosuppressive tumor microenvironments, and the development of noninvasive imaging techniques to assess cell trafficking and tumor penetration. There is also a need for studies defining biomarkers or correlates of treatment efficacy for cell-based therapies.

To support technical development, NCI should make available standard operating procedures (SOPs) for cell handling, shipment and storage, and analytical assays, as well as master specifications for cell therapy-related products. These validated guidelines would help to reduce inconsistencies across products manufactured at different sites. NCI has made great progress in this area since this need was first highlighted during the 2018 workshop (see table 1) and sharing of SOPs should continue to be a priority to promote standardization in the field. Given the strong relationship NCI has with the FDA, NCI should also work with FDA regulators, as well as the National Institute of Standards and Technology, to harmonize product characteristic specifications.

At the 2018 workshop, investigators emphasized the need for centralized GMP manufacturing for cellular therapies and viral vectors. Since then, NCI has established GMP manufacturing capability at the Biopharmaceutical Development Program (BDP) at Frederick National Laboratory for Cancer Research, allowing NCI to support cell therapy clinical trials for intramural and extramural researchers (see table 1). The BDP also has the capability to produce GMP viral vectors for cell transduction. These resources can be accessed through the NCI Experimental Therapeutic Program, open to domestic and international researchers in academia, non-profit, government and industry (https://next.cancer.gov/). In addition, investigators seeking clinical trial support as well as product manufacturing can apply to the NCI Cancer Therapy Evaluation Program (https://ctep.cancer.gov) for acceptance into a clinical trials network such as the Experimental Therapeutics Clinical Trials Network.

The ongoing demand for GMP manufacturing services was emphasized at the 2020 workshop, highlighting the need for NCI to continue expanding production of GMP vectors, cell products, and other reagents. With the growing interest in non-viral gene editing technologies, there is also a demand for CRISPR-based gene editing capability. Furthermore, 2020 workshop subjects identified the FDA-required QC testing on vectors and cell products to be a significant time and cost burden. NCI could address this challenge by making QC testing services available to investigators through the BDP. NCI could also help expand the skilled technical workforce required for cell therapy manufacturing by providing specialized training (see box 1).

### Recommendations for clinical research and the regulatory process

Both NCI workshops highlighted the advantage of multicenter, harmonized clinical trials of cellular products to efficiently translate preclinical findings to clinical trial settings. However, there is limited support for early-phase proof of concept trials and a lack of opportunity for innovation in trial design. NCI can assist the research community by supporting small adaptive clinical trials, including studies assessing pre-conditioning regimens, targeting multiple tumor antigens, and combining cell therapy with immune modulators. Establishing a framework and support for ‘Proof of Principle’ or ‘Window of Opportunity’ clinical trials for solid tumors will provide the fundamental basis to expand these approaches in larger trials and ultimately establish clinical benefit. This framework may engage established cooperative groups or new networks focused on pursuing high priority ACT approaches in solid tumors. NCI can also promote standardization in cell therapy clinical trials by establishing clinical trial protocol templates relevant to ACT for IND submissions, and facilitate a dialog with the FDA to reevaluate required testing, streamline the IND submission process, and reduce cost to investigators (see box 1).

## Conclusion

The NCI workshops on cell-based immunotherapy for solid tumors successfully identified key obstacles hindering the progress of cell-based immunotherapy and led to practical recommendations for how NCI can contribute to the advancement of this promising field. Investigators face a wide range of challenges, ranging from scientific and technical questions to clinical and regulatory barriers. NCI could act to address scientific knowledge gaps through targeted support for high-priority research areas, improve the technical process by providing standardized manufacturing procedures, and help drive early-phase proof of concept clinical trials. Through these efforts, NCI can be a source of guidance and support to the immunotherapy research community as investigators strive to improve cell-based immunotherapy treatment options and to apply these strategies to patients with advanced solid tumors.

## Data Availability

Data sharing not applicable as no datasets generated and/or analyzed for this study.
