# Supplementary material for: Challenges and next steps in the advancement of immunotherapy: summary of the 2018 and 2020 National Cancer Institute workshops on cell-based immunotherapy for solid tumors
Source: J Immunother Cancer. 2021 Jul 15;9(7):e003048. doi: 10.1136/jitc-2021-003048 (PMC8286786; doi:10.1136/jitc-2021-003048)
Supplement: Supplementary data [file jitc-2021-003048supp001.pdf]

Supplementary Table 1: 2018 Workshop Sessions

Workshop Co-Chairs:

Marcela Maus (Harvard Medical School and Massachusetts General Hospital) and Terry Fry (University of Colorado School of Medicine)

Session 1: Clinical Perspective

Speaker	Institution	Topic
Malcolm Brenner	Baylor College of Medicine	Keynote Presentation - Bit by Bit: Putting It Together
Steven Rosenberg	National Cancer Institute	Targeting Unique Somatic Mutations for the Immunotherapy of Solid Epithelial Cancers
Christian Hinrichs	National Cancer Institute	TCR Gene Therapy for Epithelial Cancers
Terry Fry	University of Colorado	Successes and Challenges with CAR-T Cell Therapy for Hematologic Malignancies
Cameron Turtle	Fred Hutchinson Cancer Research Center	Insights into Toxicities of CD19 CAR-T Cells
Marcela Maus	Harvard Medical School and Massachusetts General Hospital	Opportunities and Challenges in Developing CAR-T Cells for Solid Tumors
Panel Discussion		

Session 2: State of the Science

Speaker	Institution	Topic
Steven Albelda	University of Pennsylvania	CAR-T Cell Therapy for Solid Tumors: A Work in Progress
Stephen Gottschalk	St. Jude Children's Research Hospital	Target antigen selection for solid tumor CAR T-cell therapy
Michael Nishimura	Loyola University of Chicago	An NIH Intramural/Extramural Collaboration to Target Renal Cell Carcinoma with TCR Transduced Autologous T Cells
Renier Brentjens	Memorial Sloan Kettering Cancer Center	Moving CARs Forward: Next Generation CAR-T Cells
Travis Young	California Institute for Biomedical Research	Switchable CARs: Targeting More Than One Antigen
Dan Kaufman	University of California, San Diego	Engineered Human Pluripotent Stem Cell-derived NK Cells with Improved Anti-Tumor Activity
Panel Discussion		

Session 3: Technological Challenges for Cell Therapy

Fogli LK, *et al. J Immunother Cancer* 2021; 9:e003048. doi: 10.1136/jitc-2021-003048

Speaker	Institution	Topic
David Stroncek	NIH Clinical Center	Overview of Steps in Manufacturing Genetically Engineered T Cells
Kenneth Cornetta	Indiana University	Replicating Virus Testing in Integrating Vectors: So Far So Good
Fyodor Urnov	Altius Institute for Biomedical Sciences	Genome Editing in the Clinic for Cancer Indications: Status of the Field and General Considerations for IND-enabling Safety and Efficacy Studies
Chantale Bernatchez	University of Texas MD Anderson Cancer Center	Challenges of Expanding Tumor Infiltrating Lymphocytes (TIL) from Solid Tumors
Isabelle Riviere	Memorial Sloan Kettering Cancer Center	Challenges in CAR-T Cell Manufacturing
Lisa Butterfield	Parker Institute for Cancer Immunotherapy and University of California, San Francisco	Cellular Product Release and Potency Testing
Nirali Shah	National Cancer Institute	CAR-T Cell Therapy: Going Beyond the Test Drive
Panel Discussion		

Session 4: FDA and Industry Perspective

Speaker	Institution	Topic
Ke Liu	Food and Drug Administration	Cell-based Immunotherapy for Solid Tumors: US FDA Regulatory Perspectives
Thomas Finn	Food and Drug Administration	Regulatory Considerations for Cell and Gene Therapy Manufacturing
Jake Reder	Celdara Medical and Giesel School of Medicine at Dartmouth	Cell-based Immunotherapy for Solid Tumors: The Role of Small Business
Jason Cristofaro	National Cancer Institute	Partnering with the NCI: Intellectual Property Issues
Panel Discussion		

Video of the 2018 workshop can be accessed at:

Workshop Day 1 (December 10, 2018): https://videocast.nih.gov/summary.asp?Live=29065&bhcp=1

Workshop Day 2 (December 11, 2018): https://videocast.nih.gov/summary.asp?Live=29069&bhcp=1

Fogli LK, *et al. J Immunother Cancer* 2021; 9:e003048. doi: 10.1136/jitc-2021-003048

Supplementary Table 2: 2020 Workshop Sessions

Workshop Co-Chairs:

Crystal Mackall (Stanford University) and Marcela Maus (Harvard Medical School and Massachusetts General Hospital)

Session 1: Choosing the Target: Specificity and Toxicity

Chair: Madhav Dhodapkar (Emory University)

Speaker	Institution	Topic
Phil Greenberg	Fred Hutchinson Cancer Research Center	Selecting and Validating Targets, Isolating High Affinity TCRs, & Engineering T Cells That Can Be Effective in Therapy
Kole Roybal	University of California, San Francisco	Development of Combinatorial Antigen Sensing T Cell Therapeutics for Solid Tumors
Christopher Klebanoff	Memorial Sloan Kettering Cancer Center	Targeting Solid Malignancies With "Public" Neoantigen-Specific T Cell Receptors
Christian Hinrichs	National Cancer Institute	Safety and Clinical Activity of Engineered TCR-T Cells for HPV-Associated Cancers
Panel Discussion with all speakers<br><br>Additional panelists:<br>Laronna Colbert (Food and Drug Administration)<br>Wendell Lim (University of California, San Francisco)		

Session 2: Reaching the Target: Modulating the Tumor Microenvironment

Chair: Yvonne Chen (University of California, Los Angeles)

Speaker	Institution	Topic
Greg Delgoffe	University of Pittsburgh	Feast to Famine: Preparing Therapeutic T cells for Intratumoral Function through Metabolic Reprogramming
Renier Brentjens	Memorial Sloan Kettering Cancer Center	Armored CAR T Cells and Solid Tumor Malignancies
Catherine Bollard	Children's National Research Institute and George Washington University	Enhancing Cell Therapies by Overcoming TGFbeta in the Tumor Microenvironment
Antoni Ribas	University of California, Los	Does It Make Sense to Add Immune Checkpoint Blockade to Adoptive Cell Transfer Therapies?

	Angeles	
Panel Discussion with all speakers<br><br>Additional panelists:<br>Cliona Rooney (Baylor College of Medicine)<br>Michael Hudecek (University of Würzburg)		

Session 3: Immune Cell Fitness and Persistence

Chair: Alex Marson (University of California, San Francisco)

**Speaker**	**Institution**	**Topic**
Carl June	University of Pennsylvania	The Tug-o-War Between CAR T and Solid Tumors
Stanley Riddell	Fred Hutchinson Cancer Research Center	Driving CAR T Cells into Solid Tumors Without Exhaust(ion)
Andrea Schietinger	Memorial Sloan Kettering Cancer Center	Decoding T Cell Dysfunction in Solid Tumors
Alex Marson	University of California, San Francisco	Decoding and Reprogramming T Cell Fitness with CRISPR
Panel Discussion with all speakers<br><br>Additional panelists:<br>Ananda Goldrath (University of California, San Diego)<br>Wendell Lim (University of California, San Francisco)		

Session 4: Alternative Approaches: Looking Beyond Traditional CAR-T Cells

Chair: Tonya Webb (University of Maryland)

**Speaker**	**Institution**	**Topic**
Steven Rosenberg	National Cancer Institute	Cellular Therapy of Solid Epithelial Cancers Using Naturally Occurring or Genetically Modified Anti-Tumor T Cells
Lili Yang	University of California, Los Angeles	Looking Beyond Traditional CAR-T Cells: Unconventional T Cells, Stem Cell Engineering, and an Off-The-Shelf Cell Therapy
Pawel Kalinski	Roswell Park Comprehensive Cancer Center	Dendritic Cells: Vaccines, Modulation of Tumor Microenvironment and Adoptive T Cell Therapies
Lawrence Lum	University of Virginia	Clinical and Immunologic Effects of Bispecific Antibody Targeted T Cells for Metastatic Breast and Pancreatic Cancer

Panel Discussion with all speakers<br><br>Additional panelists:<br>Dan Kaufman (University of California, San Diego)

Session 5: Cell Product Manufacturing and Characterization

Chair: Bruce Levine (University of Pennsylvania)

Speaker	Institution	Topic
Samantha Maragh	National Institute of Standards and Technology	The Role of Standards in Supporting Cell-Based Therapies & Genome Editing Technology
Bruce Levine	University of Pennsylvania	Design, Engineer, Validate, Build, Test: Manufacturing the Next Generation of Cell Therapies Targeting Solid Cancers
Christine Brown	City of Hope	Manufacturing Matters: Population, Phenotype & Potency
Barbra Sasu	Allogene Therapeutics	Considerations for Manufacturing an Allogeneic Cell Product
Panel Discussion with all speakers<br><br>Additional panelists:<br>Raj Puri (Food and Drug Administration)<br>Tal Salz (Food and Drug Administration)<br>Anthony Welch (National Cancer Institute)		

Session 6: Cell Therapies: The FDA Perspective

Chair: Elad Sharon (NCI)

Speaker	Institution	Topic
Alyssa Galaro	Food and Drug Administration	Regulatory Perspective on the Preclinical Development of Cell-Based Immunotherapies
Irina Tiper	Food and Drug Administration	Identification and Assessment of Critical Quality Attributes for Cell-Based Therapies
Yuxia Jia	Food and Drug Administration	FDA Clinical Assessment for First-In-Human Studies of Cell Therapy in Solid Tumors
Panel Discussion with all speakers<br><br>Additional panelists:<br>Tal Salz (Food and Drug Administration)<br>Raj Puri (Food and Drug Administration)		

Fogli LK, *et al. J Immunother Cancer* 2021; 9:e003048. doi: 10.1136/jitc-2021-003048

Elad Sharon (National Cancer Institute)

Keynote Talks

Marcela Maus (Harvard Medical School and Massachusetts General Hospital) – Emerging Challenges and Strategies with CAR T cells

Crystal Mackall (Stanford University) – Approaches to Augment Potency of CAR T Cell Therapies

Video of the 2020 workshop can be accessed on the meeting website:

https://events.cancer.gov/dctd/celltherapyconf
